# 43 Mapping the Hypermetabolic Response in Burn Patients

**DOI:** 10.1093/jbcr/irac012.046

**Published:** 2022-03-23

**Authors:** Carly M Knuth, Zachary Ricciuti, Dalia Barayan, Sarah Rehou, Marc G Jeschke

**Affiliations:** University of Toronto, Toronto, Ontario; Sunnybrook Research Institute, Toronto, Ontario; University of Toronto, Toronto, Ontario; Sunnybrook Health Sciences Centre, Toronto, Ontario; U of T, Toronto, Ontario

## Abstract

**Introduction:**

Hypermetabolism, characterized by drastic increases in whole-body catabolism and resting energy expenditure (REE), is a hallmark response to a severe burn injury. This is believed to be driven in part by alterations in adipose tissue metabolism. We proposed to define the hypermetabolic response in adipose tissue from burn patients and create a roadmap of markers indicative of hypermetabolism to improve prognosis. We hypothesized that catabolic markers, such as uncoupling protein-1 (Ucp1) and growth differentiation factor-15 (Gdf15), would positively correlate with increasing days post-burn and REE.

**Methods:**

Adult burn patients (n=65) admitted to our burn center between 2011—2019 were included in this study. Subcutaneous white adipose tissues (sWAT) from the site of injury (n=85) and plasma were collected from severely burned patients ( ³20% total body surface area). Gene expression and circulating cytokine levels were measured by RT-qPCR and multiplex assays, respectively.

**Results:**

We found a significant correlation between increasing Ucp1 gene expression and days post-burn (p< 0.0001). Moreover, when samples were stratified into acute (1-3 days post-burn), moderate (4-9 days post-burn), and long-term ( >10 days post-burn) timepoints, a significant increase in Ucp1 gene expression was detected only in adipose tissues from long-term time points in comparison to non-burned control tissues (p< 0.01). However, we found that REE remained stagnant throughout hospital stay after a burn injury in our patient cohort. Thus, we did not detect a significant correlation between Ucp1 gene expression and REE. Further, while Gdf15 expression was most pronounced, albeit statistically insignificant, during the moderate timepoints, we did not detect any significant differences when correlated with days post-burn. Additionally, we determined that circulating levels of IL-6, IL-10, and monocyte chemoattractant protein-1 (MCP-1) were greatly elevated within the first seven days post-burn and gradually decreased over time, while vascular endothelial growth factor (VEGF) concentrations followed a similar pattern to Ucp1 gene expression.

**Conclusions:**

While Gdf15 expression may not accurately reflect catabolism in the adipose tissues of burn patients, Ucp1 gene expression may be used as a marker indicating a peak hypermetabolic period after ten days post-burn. This may also be reflected by circulating concentrations of VEGF. Moreover, IL-6, IL-10 and MCP-1 may be used as early determinants before the onset of hypermetabolism.

